# Hydrated Chloral—Chloral Hydrate

**Published:** 1893-12

**Authors:** John R. Anderson

**Affiliations:** Phoenix Hotel, Sixth Street, Louisville, Ky.


THE JOURNAL

OF

COMPARATIVE MEDICINE AND

VETERINARY ARCHIVES.

VOL. XIV. DECEMBER, 1893. No. 6.

### HYDRATED CHLORAL.—CHLORAL HYDRATE.

### CHLORAL.

BY JOHN R. ANDERSON, D.V.S.

To the veterinary practitioner this potent chemical compound is of far more value as a therapeutical agent than to the regular physician, as its administration to animals is unattended by any evil results, except when given in enormous quantities. Its field of utility is broad, and it will, with further investigation, prove, no doubt, to be of far more value than has as yet been demonstrated. Chloral appears in commerce in two forms; as a white translucent flake, and in transparent granules, both having a pungent nauseant taste. It is soluble in one and one-half times its weight in cold water at, say, 50 degrees Fahr. Much more so in hot water, but should never be dissolved in a medium warmer than 60 degrees Fahr. Alcohol, ether and glycerine also prove solvents for it, but not in so large quantity.

Chloral should never be incorporated with any alkaline substance as they decompose it, the resultant being chloroform and formic acid. Fixed oils prove fair solvents and good vehicles for its internal administration and local application. A 50 per cent. solution (watery) of hydrated chloral will preserve anatomical and morbid specimens indefinitely. When equal parts of chloral hydrate and gum camphor are rubbed together in a mortar, or they are mixed in fine particles and allowed to stand in a close-stoppered jar, a clear oily liquid results.

An equal part of tincture of iodine added to this mixture cannot be too strongly commended when applied to erysipelatous swellings in the horse before suppuration takes place; always clipping the hair off, however, before applying and painting the parts daily, and confining the patient's head so that he cannot get at it with his mouth. It has no merit, I find, after the suppurative stage has been fully established.

One-half to one ounce of this oil proves most effectual in certain forms of non-organic visceral diseases.

Diluted with one pint of oil (raw linseed preferable), nothing is more effectual in simple spasmodic colic resulting from atmospheric influence, or pleuritic attacks, non-specific, in the primary stage, than six drachms hydrated chloral or one-half ounce of the camphorated chloral oil.

In the lower animals chloral exerts but little or no influence upon the cerebrum, as it does in man; its hypnotic influence can be said to be entirely lost. With rare exception we find a patient susceptible to its hynotizing effects. It acts feebly but certainly upon the cerebellum, and more perceptibly upon the medulla oblongata. Its influence is expended upon the nerve centres of the spinal cord almost in toto.

If an almost lethargical dose of chloral hydrate (say, an ounce to an ounce and a half) be given a horse suffering from abdominal pains, there first occurs a complete cessation of the pain; secondly, a decided disturbance of coördination, and thirdly, a suspension of voluntary motor power. This condition lasts from one to two hours, when the power of movement returns, and later a reëstablishment of coördination. When given in full therapeutical doses, it is followed, in from thirty to forty minutes, by a gradual diminution, and finally by a complete disappearance of all visceral pain; this is specially true if the lesion lies in the large intestine. The heart's action may drop a few beats, but this does not occur often; the volume is usually slightly diminished instead. Respiration is affected proportionately with the heart. No alteration of the body heat is perceptible, nor does there occur any disturbance to peristalsis. Elimination of the drug takes place chiefly by the kidneys, partially through the skin. The camphorated chloral oil with one-third part olive or cotton-seed oil added, gives great relief when applied to painful wounds of the feet in horses and cattle.

At the onset of enteritis, peritonitis, cystitis, metritis, and in fact all painful affections within the cavities which are of an inflammatory type, functional and dependant upon local causes, the symptoms are greatly relieved and sometimes cut short by a full dose of chloral hydrate.

After the stage of effusion it is of no value, however, except in the ratio as it temporarily relieves pain. In those cases of muscular rigidity, from clonic spasms, resulting from uterine irritation (so-called) and reflection, which we so often meet with in bitches after whelping, there is no agent or agents in the whole pharmacopœia which gives such prompt, entire and permanent relief as does a full dose of chloral hypodermically administered. The quantity regulated according to body weight of the dog. Six to ten grains for a 10 to 20 pound dog; 10 to 15 grains for a 20 to 60 pound dog; 15 to 20 grains for a 60 to 125 dog, so on. I once gave a bitch weighing 128 pounds, in 20 grain doses, 80 grains within 30 minutes. But the patient was extremely rigid, and had been for 24 hours previous to her being brought to me. The hypnotic influence of the drug in this case was manifested, but not so profound that it was difficult to arouse the bitch from sleep, which by the way resembled natural sleep more closely than that produced by other drugs. Strychnia poisoning, so common to dogs, can be most certainly and successfully combated by this agent.

The toxicological effects of strychnia are antagonized through their entire range by chloral hydrate, and it can be stated with impunity that in strychnia poisoning of dogs, if there is a vestige of life left, with the hypodermatic injection of a sufficient quantity of chloral, the patient cannot, will not die. In my practice it has occurred that the initial dose was necessarily large for the pound weight of the dog, but by carefully watching the effects of the drug and being guided therefrom as to each successive dose, the results obtained have been without an exception gratifying in the extreme.

Six or seven years ago, while in conversation with a man who had been engaged in shipping as an attendant with horses to and from the central blue grass region of Kentucky to New York and California, he described a condition as occurring frequently among horses in his care, which he was wont to call insanity from fright. He said after being loaded on the cars and fairly on their journey, the subjects in question would become restless, break out in a profuse perspiration, tremble from head to foot, grow violent and finally die with their jaws locked, and the muscles of their bodies as rigidly contracted as if they had tetanus, unless they were unloaded at the first station and something done for them. In questioning him I learned that in every instance those affected were horses that had been taken up off of grass pastures only a few days before shipping, or else they were brood mares shipped to or from the stud, and that they were at the time of the attack well into the œstral period. It was not until 1889, however, that a case of this character came under my observation The veterinary surgeon in charge was treating it with cold water showers over the body and some medication bv the mouth. The treatment was unsuccessful, as others thereafter proved. In 1890 I was asked to attend a brood mare that had been led a distance of 4 or 5 miles to be bred. The superintendent stated that she had gone along quiet, but on reaching the farm they were unable to get the bit from her mouth, and at that moment she was trembling, sweating, and could not stand still.

On reaching the place, this case was a most urgent one. She had been turned loose into a box stall; her jaws were locked, every muscle of the body on the surface could be outlined; the successive contractions of the muscles of the limbs were so violent, and co-ordination so disturbed, that it was with the greatest difficulty that she was enabled to maintain the standing posture. Her head was protruded and the membrana nictitans was so drawn as to completely cover the globe. Drenched with perspiration she was a miserable picture of agony. She could not remain quiet one moment; and with difficulty and the loss of two syringe needles I succeeded in getting chloral hydrate, one half ounce in one ounce of water (cold), under the skin. Within 15 minutes she was able, with some effort, to stand still; 20 minutes more and she could direct her steps; 10 minutes more the membrana nictitans had gained its normal position; and in one hour and five minutes not a symptom was left to betray the fearful condition which had existed a little more than an hour previous. This subject weighed about 950 pounds.

Since that date the number I have treated will not fall far short of a half hundred, and it has been necessary with a few to give six drachms before obtaining the desired result; but I have always been rewarded by success. Abscesses invariably follow the hypodermatic use of chloral, but they heal rapidly, so soon as the sack is emptied. In localities where the horse reaches 14 to 20 hundred weight, I think it safe for practitioners to make the initial dose from six to eight drachms, hypodermically, according to the severity of the attack. For the benefit of breeders and shippers to any distance, who are not in a position to secure the services of a qualified man, I would suggest that before starting valuable mares or horses, just from the grass fields, on long journeys on the cars, they should provide their attendant with a cheap hypodermic syringe and one or two one ounce bottles containing chloral hydrate, one-half ounce and water one ounce, with instructions how to insert the needle into the skin. The treatment is a safe, simple and certain one, unattended by any evil results, save the abscesses, and they are of no consequence in reality, and can be attended to so soon as the animals reach their destination. Always insert into the skin of the breast, low down, as the abscesses are not so large when the point of origin is located dependently and they drain much more easily.

Three hundred and twenty grains of chloral swallowed, dissolved with two ounces of water in a graduating glass, by a man who was a perfect athlete, with suicidal intent, did not prove fatal. Profound hypnotism occurred in twenty minutes. The subject came into my room immediately after taking the dose and lost consciousness shortly thereafter. I gave him, hypodermically, ¼ grain of strychnina sulphate and sent in haste for an M. D., who responded promptly. We learned the quantity he had taken and gave ½ grain more sulphate of strychnine. Oxygen gas was given by inhalation and the faradic current applied continuously. The dose was swallowed at 7 o'clock A. M. It was 3 o'clock the next morning when he aroused, which he did of his own accord, and was, to all appearances, as if he had awakened from a natural deep sleep. He sat up in bed without assistance, conversed with me about what he had done for five or ten minutes, lay down fell asleep again, and awoke at 6.30 A. M.

Why his œsophagus was not excoriated from contact with so concentrated a solution is difficult to understand. His stomach being filled with beer, as he stated afterwards, accounts for no injury being done to that organ. Frequently during the twenty-four hours it was necessary to introduce the catheter into his bladder as he could not void his urine. He complained of nothing else, nor of any ill state of being during his recovery.

The case is an instructive one, since we know that there are cases on record, in man, in which death was produced by twenty grains of hydrated chloral, and the usual quantity given to man therapeutically is ten to fifteen grains.

Phœnix Hotel, Sixth Street,
Louisville, Ky.

